# Towards using scattered PET emission data for reconstruction of attenuation map in PET/MRI

**DOI:** 10.1186/2197-7364-1-S1-A34

**Published:** 2014-07-29

**Authors:** Yannick Berker, Volkmar Schulz

**Affiliations:** Department of Physics of Molecular Imaging Systems, Institute for Experimental Molecular Imaging, RWTH Aachen University, Aachen, Germany; Philips Technologie GmbH Innovative Technologies, Research Laboratories, Aachen, Germany

Despite promising advances, attenuation correction of PET data remains challenging in PET/MRI. Known approaches to estimating attenuation maps from PET emission data take into account only true coincidences and fail to completely determine the attenuation map without prior information. Even TOF PET emission data determines the attenuation sinogram up to an unknown constant only. We propose to extract information from scattered PET coincidences to fully determine the attenuation map.

Scatter-to-attenuation reconstruction has been approximated by a proof-of-concept scatter-to-attenuation back-projection. The latter is based on determining possible scattering locations from energy measurements (via Compton scattering angles) and summing contributions from scattered coincidences in image voxels. Contributions are weighted based on geometrical considerations and various corrections derived from a scatter-measurement equation similar to the single-scatter-simulation formula; this equation represents a weighted Radon transform on surfaces in curvilinear coordinates. The back-projection technique was evaluated with data from GATE simulations of various activity and attenuation distributions in a clinical scanner model. Finally, the potential of the back-projection operator for determination of a unique attenuation map has been evaluated in an iterative scheme for small-scale problems.

Scatter-to-attenuation back-projection of coincidences with scattered photon energies in the range of 248–478 keV are shown for general cases as well as for highly degenerate cases with perfect spherical symmetry of attenuation and activity distributions. The parameter-space trajectory followed by the iterative scheme shows that the proposed scatter-to-attenuation back-projection can be used to estimate true attenuation values despite an initially unknown scaling factor.

Scattered coincidences provide information that may complement existing reconstruction approaches. The proposed scatter-to-attenuation back-projection may constitute a missing piece for resolving ambiguities in the simultaneous reconstruction of activity and attenuation. Lacking an analytical reconstruction procedure, iterative techniques need to be developed to fully integrate information from true and scattered coincidences.

